# A metabolomic study of* Gomphrena agrestis* in Brazilian Cerrado suggests drought-adaptive strategies on metabolism

**DOI:** 10.1038/s41598-021-92449-9

**Published:** 2021-06-21

**Authors:** Geraldo Aclécio Melo, Ilka Nacif Abreu, Maíra Baista de Oliveira, Ilara Gabriela Frasson Budzinski, Lucinélia Vieira Silva, Marcio Antônio Silva Pimenta, Thomas Moritz

**Affiliations:** 1 Departamento de Biologia Geral, Universidade Estadual de Montes Claros, Montes Claros, MG 39401-089 Brazil; 2 Department of Forest Genetics and Plant Physiology, Swedish University of Agricultural Sciences, Umeå Plant Science Centre, 901 83 Umeå, Sweden; 3 Novo Nordisk Foundation Center for Basic Metabolic Research, Faculty of Health and Medical Sciences, University of Copenhagen, Copenhagen, Denmark

**Keywords:** Plant sciences, Plant physiology, Plant stress responses

## Abstract

Drought is the main factor that limits the distribution and productivity of plant species. In the Brazilian Cerrado, the vegetation is adapted to a seasonal climate with long- and short-term periods of drought. To analyze the metabolic strategies under such conditions, a metabolomic approach was used to characterize *Gomphrena agrestis* Mart. (Amaranthaceae) a native species that grows under natural conditions, in a rock-field area. Roots and leaves material from native specimens were sampled along different seasons of the year and LC–MS and GC–MS analyzed for multiple chemical constituents. The datasets derived from the different measurements were combined and evaluated using multivariate analysis. Principal component analysis was used to obtain an overview of the samples and identify outliers. Later, the data was analyzed with orthogonal projection to latent structures discriminant analysis to obtain valid models that could explain the metabolite variations in the different seasons. Two hundred and eighty metabolites were annotated, generating a unique database to characterize metabolic strategies used to cope with the effects of drought. The accumulation of fructans in the thickened roots is consistent with the storage of carbons during the rainy season to support the energy demand during a long period of drought. The accumulation of Abscisic acid, sugars and sugar alcohols, phenolics, and pigment in the leaves suggests physiological adaptations. To cope with long-term drought, the data suggests that tissue water status and storage of reserves are important to support plant survival and regrowth. However, during short-term drought, osmoregulation and oxidative protection seems to be essential, probably to support the maintenance of active photosynthesis.

## Introduction

The Cerrado (Neotropical savanna) covers about 2 million km^2^ of Central Brazil and represents about 23% of the land surface of the country^1^. It is one of the richest and most diverse savanna worldwide and considered one of 34 global hotspots for biodiversity^2^. A striking feature of the Cerrado is the seasonal climate with a dry season that extends from May to September and a rainy season with precipitation ranging from 800 to 1800 mm (90% between October and April)^3^. In the rock-field formations of the Cerrado, the soil is shallow and sandy with low water-holding capacity^4–6^. The rock-field vegetation is characterized by herbaceous shrubs, with a predominance of endemic and rare species with nutritional and biological specializations to acquire resources from the soil and survive under high irradiance, strong winds, and low water availability in the soil^5,7,8^. Because of the specific climate (long and short periods of drought), soil physical characteristics, and adapted native flora, the rock fields of Cerrado are a natural resource for studying plant traits and responses under limited water conditions.

All plant species have varied tolerance or resistance to drought, depending on their phenological, morphological, physiological, and biochemical characteristics. All these adaptive mechanisms allow the plants to escape or tolerate the stress condition by either avoiding, reducing, or resisting dehydration^9–11^. The drought stress in plants occur when either the water supply to their roots becomes limiting or when the transpiration rate becomes too intense that the system can not reach a balance^9^. At these points, the stress starts affecting the water functions, early, reducing the water potential and turgor, what lead to reduction in cell enlargement and culminating in reduced growth and the potential of growth. Subsequently, passive or active stomata closure will limit CO_2_ supply for photosynthesis^12–14^, what by itself is sufficient to constrain carbon assimilation and to starts a network of effects in process that are carbon dependent. Besides, under day light, with the reduction of the flux of electrons for carbon assimilation, it may lead to overproduction of reactive oxygen species^15,16^, that in turn will cause more constraints to the already reduced photosynthetic capacity of the plant. Depending on the intensity and duration of the stressing condition more constraints can be imposed and the plant can be put to death.

In the Cerrado rock-fields, where the species can experience water stress in different extent, it was already reported in the plants characteristics to deal with the water availability, such as the presence of aerial roots with velamen or pseudocaules that play a role in the access of moisture of dew and rain^17^. In some specie, leaves in rosette forms, as in bromeliads, allow water retention^18^ and in many other plants water-secreting structures and trichomes are also common, and may play a role in atmospheric water uptake^19^. Thickened underground organs that accumulates reserves, mainly carbohydrates, are also common in shrubs and herbs and may play an important role in water storage and maintenance of the water status^20–23^. The type and content variation along season of fructans, are mentioned to be important in the osmoregulation process^20,22^. Also, it is a significant source of nutrients and carbon to be used during regrowth after drought^23,24^.

Global climate changes have led to concerns regarding the effects of drought on plant development and agricultural and natural ecosystems^25,26^. Changes in temperature and rainfall patterns could result in limited water for plant growth in some regions, especially agricultural lands^27,28^. Studies need to be performed to understand how known strategies were established along with plant evolution, how plants will be affected by and respond to climate change, as well as how we can use this information to develop and manage agriculture in the future, for example, develop varieties that can maintain optimum yield levels even under stress conditions and establish irrigation management^29,30^.

Here, to investigate metabolic patterns that can be associated with the drought in plants growing under rock-field conditions, several metabolomic approaches were used to characterize the metabolite profile of *Gomphrena agrestis* Mart. (Amaranthaceae), a native species^31^ along different season in 1 year. The goal questions were: (1) How is the availability of water in the soil along seasons? (2) Do the plants have the water status changed along seasons and can it be associated with the water availability in the soil? (3) What are the main changes in metabolite patterns in the plants? A database of 280 metabolites was generated, which include primary and secondary metabolites, hormones, and lipids, which was used to understand metabolic adaptations under short- and long-term periods of limited water conditions in rock-fields.

## Results

### Water status of the soil and plants

The precipitation data from the region are shown in Supplementary Table 1. From September to November (2013) and February to July (2014), great variations in rainfall were observed in the region, with the highest index in April (75 mm of rain). In February, even in middle of the rainy period, a reduction in rainfall rates was detected (13 mm in February). The past data for the region have shown lower precipitation rates in February (INMET, 2017), leading to a short dry period during the rainy season (this period is popularly called “Veranico”). In July, which is a part of the dry season, no precipitation was recorded.

**Figure 1:**
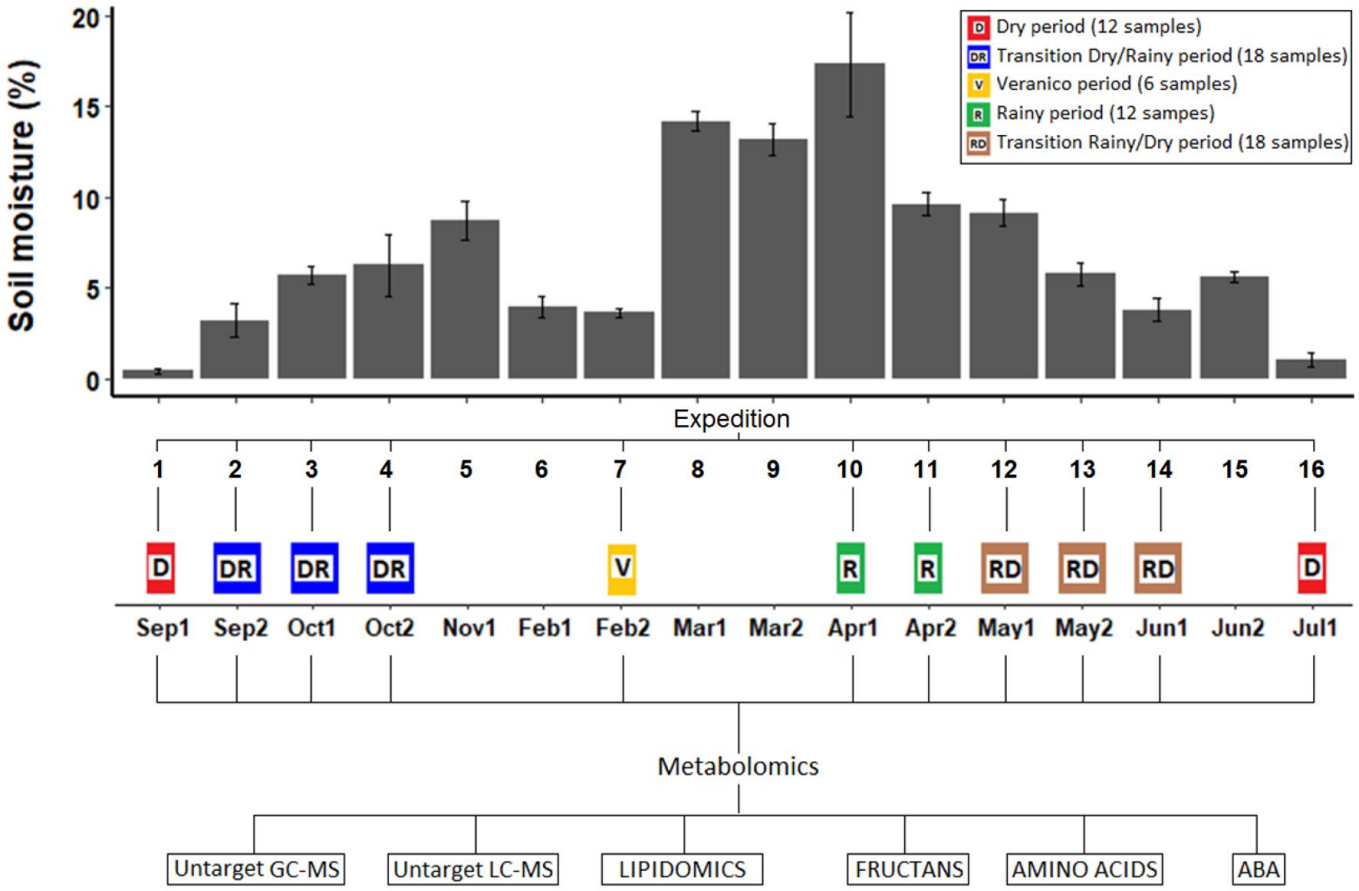
Design of sampling and sample grouping for the metabolomic analysis. The groups are as follows: D, dry period (12 samples, red color); DR, transition from dry to rainy period (18 samples, blue); V, veranico, a short dry period during the rainy period (6 samples, yellow); R, rainy period (12 samples, green); and RD, transition from rainy to dry period (18 samples, brown). Below, months of the year and the number of the sample in the corresponding month. Soil moisture was used to select samples for metabolomics.

The soil moisture was positive correlated (*p* < 0.05) with the rainfall recorded for the period and ranged from 0.4 to 17.3% (Fig. 1). Interestingly, during the rainy season (from November to April), a decrease in the percentage of soil moisture, in February, matches with the Veranico period. In the months that comprise the dry periods, soil moisture was 0.4% in early September 2013 and 1.05% in July 2014**.**

### Phenology

**Figure 2:**
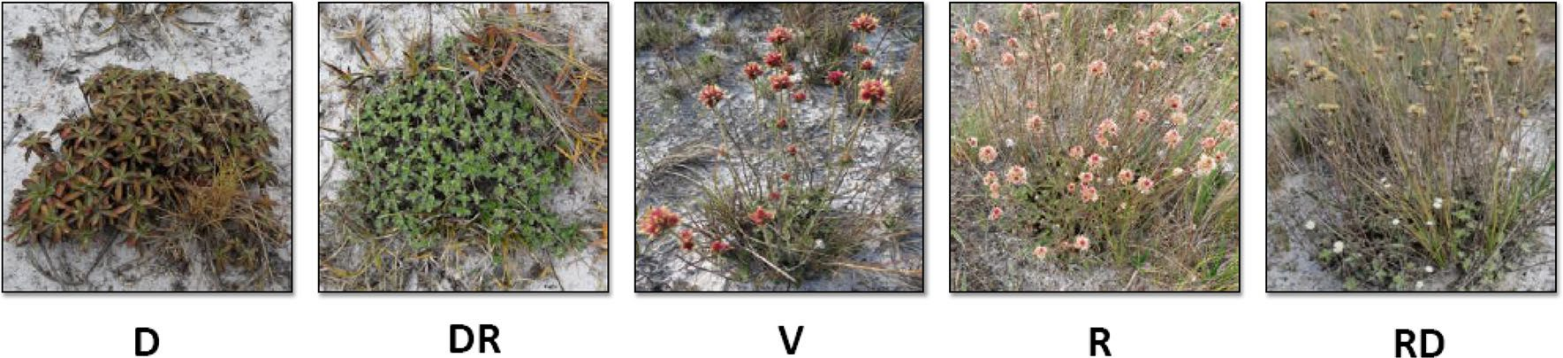
Phenological characterization of *Gomphrena agrestis* Mart. (Amaranthaceae) in a rock-field area in the dry and rainy seasons. D, dry period; RD, transition from dry to rainy period; V, veranico period; R, rainy period; and RD, transition from rainy to dry period.

The predominant phenological stage annotated for the species was compiled and represented in Fig. 2. At the end of the dry period, in early September, the plants were characterized in a dormant state, showing senescent floral branches and older leaves. In the DR period in October, the plants entered the sprouting state, with emission of new branches and leaves. In the Veranico period in February, presence of inflorescences with reddish floral parts was observed. In the rainy period in April, the fruiting phase was characterized by the presence of inflorescences with senescent and paleaceous floral parts. The fruiting phase lasted until May in the DR period, and the senescence of the floral branches was remarkable. In July, at the beginning of the dry period, the plants entered dormancy. In the dormant state, the younger leaves on the branches of the plants were still green.

### Abscisic acid content

**Figure 3:**
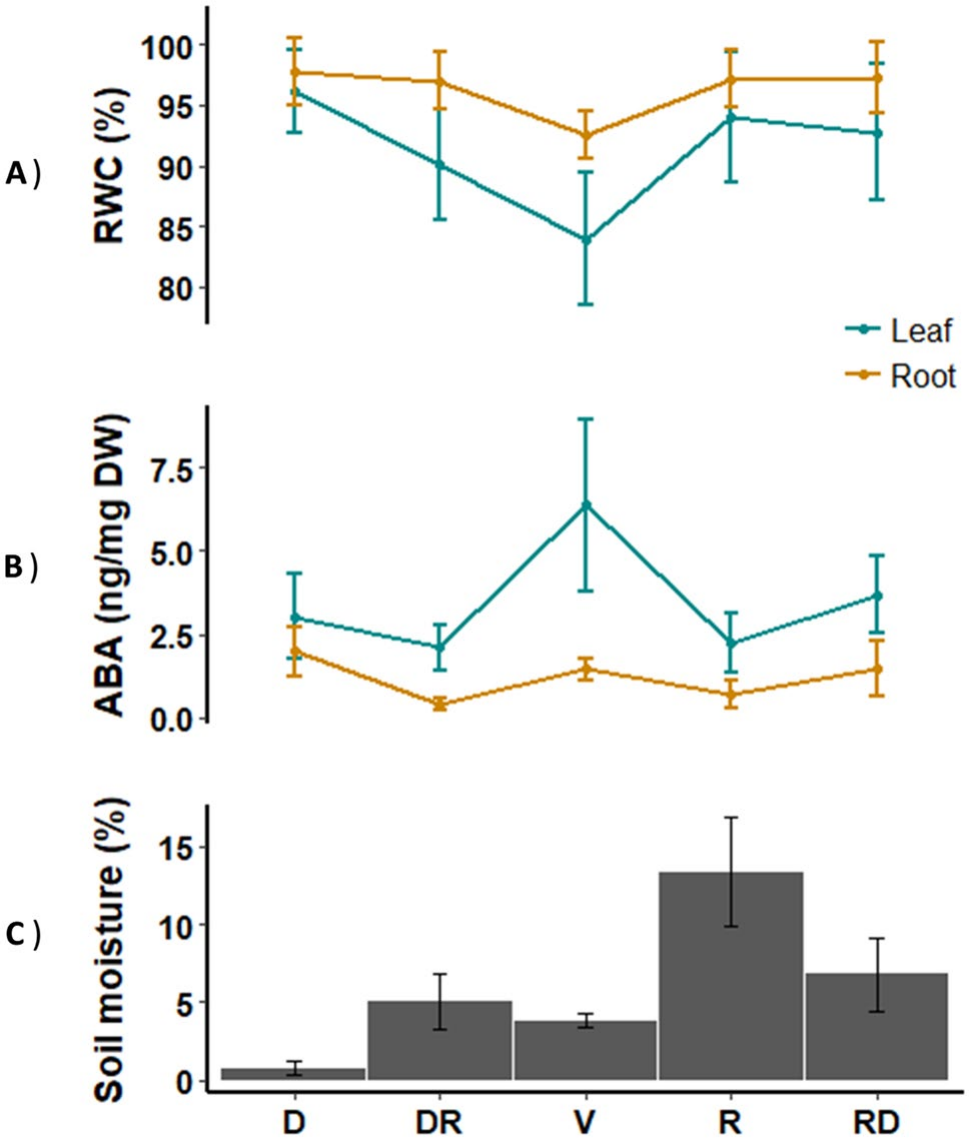
Relative water content (RWC)—(**A**) abscisic acid content (ABA)—(**B**) and Soil moisture—(**C**) in leaves and roots of *Gomphrena agrestis* Mart. in a rock-field area in the dry and rainy seasons. *D* dry period, *RD* transition from dry to rainy period, *V* veranico period, *R* rainy period, *RD* transition from rainy to dry period. Performed using R-software version 3.4.1^66^.

In Fig. 3, the relative water content (RWC), abscisic acid (ABA) content, and Sm are summarized as an average of the samples of the D, dry period; RD, transition from dry to rainy period; V, veranico period; R, rainy period; and RD, transition from rainy to dry period. No correlation was observed between Sm and leaf and root RWC values of the plants. Nevertheless, in the Veranico period, RWC of the leaves was slightly lower when there was a decrease in Sm (Fig. 3A).

Abscisic acid (ABA) content in the roots and leaves was measured because of its important role in plant responses to drought and other abiotic stresses (Fig. 3B). The general pattern of ABA accumulation was similar in the leaves and roots; however, ABA content was significantly higher in the leaves (Tukey’s test, *p* < 0.05) in the V period than in the DR and R periods.

### Metabolic profiling

**Figure 4:**
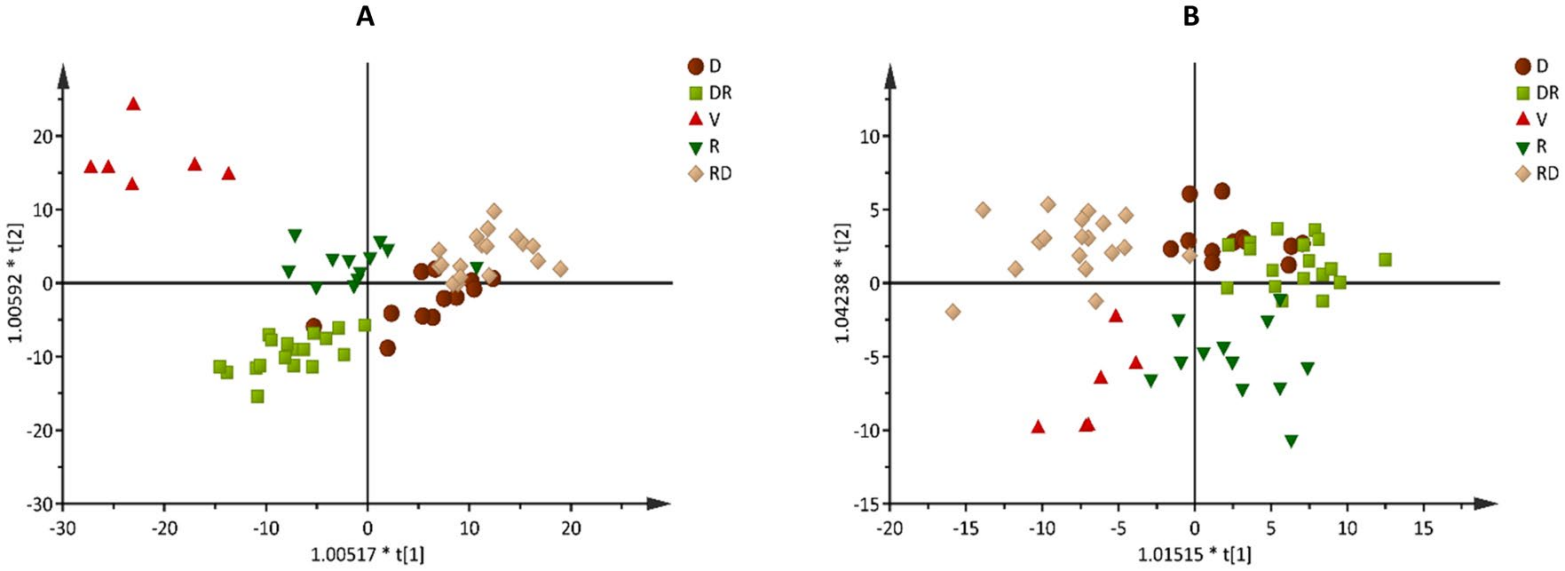
Multivariate data modelling: Score plot of orthogonal projection to latent structures discriminant analysis (OPLS-DA) performed on metabolites in leaves (**A**) and roots (**B**) of *Gomphrena agrestis* Mart. in a rock-field area in the dry and rainy seasons. Sample groups: *D* dry period, *RD* transition from dry to rainy period, *V* veranico period, *R* rainy period, *RD* transition from rainy to dry period.

**Figure 5:**
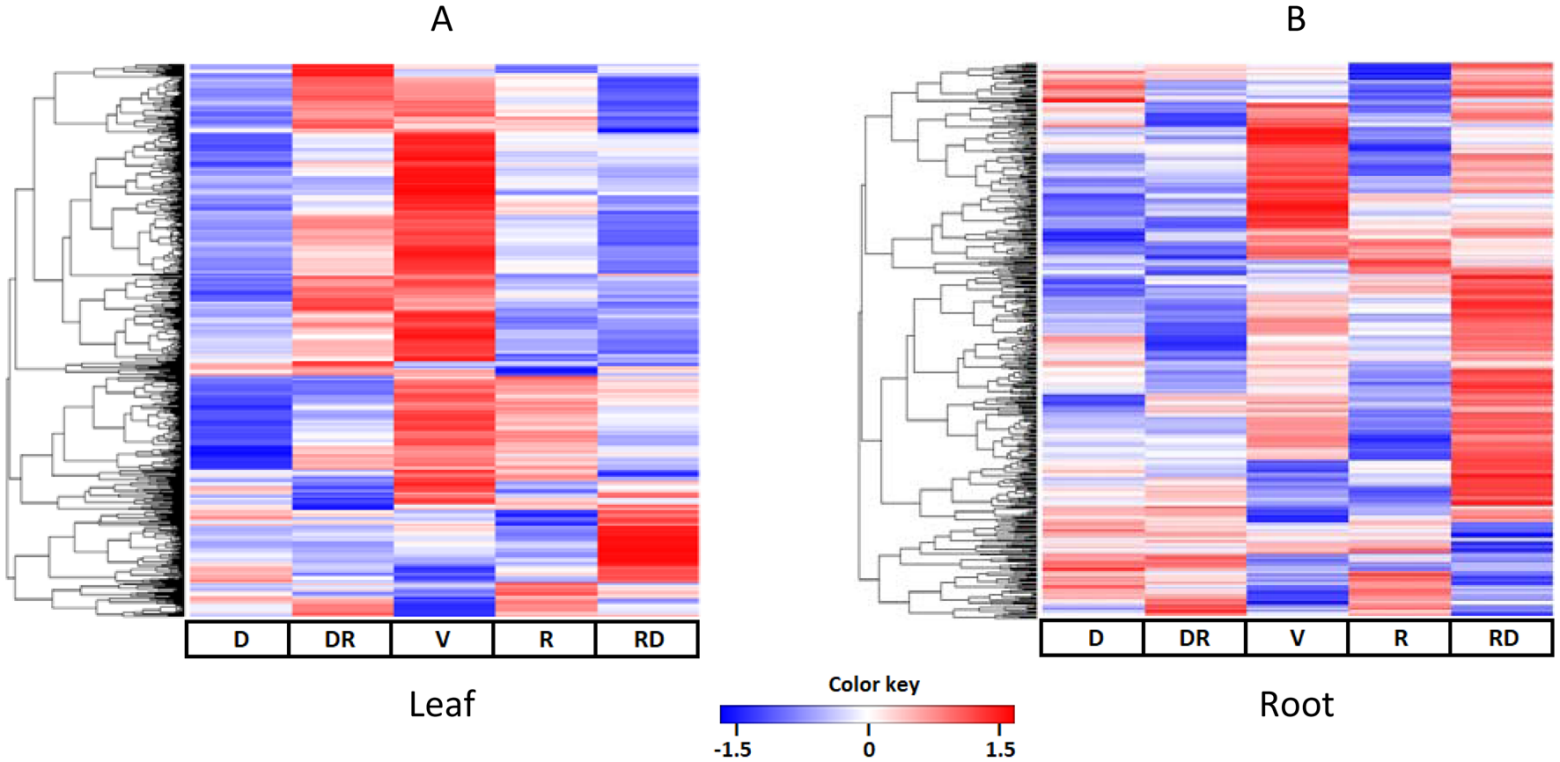
Heatmap visualization from a cluster hierarchical analysis of leaf and root metabolites of *Gomphrena agrestis* Mart. in a rock-field area in the dry and rainy seasons. *D* dry period, *RD* transition from dry to rainy period, *V* veranico period, *R* rainy period, *RD* transition from rainy to dry period. Performed using R-software version 3.4.1^66^.

To obtain information on the metabolic phenotypes of *G. agrestis* grown under natural conditions in a rock-field area of the Cerrado in the dry and rainy seasons, we performed a multi-metabolomic analysis of the leaves and roots. The datasets derived from different measurements were combined and evaluated using multivariate analysis. Principal component analysis (PCA) was used to obtain an overview of the samples and identify outliers (data not shown). Later, the data was analyzed with orthogonal projection to latent structures discriminant analysis (OPLS-DA) to obtain valid models that could explain the metabolite variations in the different seasons. From the generated loading plots, the metabolites were filtered by variable importance in the projection (VIP) (cutoff ≥ 1) and listed**.** On the basis of the selected VIP metabolites, OPLS-DA score plots of the leaves and roots are shown in Fig. 4. To validate the strategy, several pair-wise OPLS-DA models were calculated between the groups by using the selected metabolites (Supplementary Table 2). Because all comparisons resulted in valid models, the distinct metabolites were clustered, and the heatmaps (Fig. 5A,B) show a global metabolite profile of the leaves and roots of *G. agrestis* in different seasons.

The metabolites derived from the untargeted approach with GC–MS and LC–MS analyses were systematically annotated (Fig. 5A,B, as described in the “Material and methods”). The combination of targeted and untargeted approaches allowed us to have a significant coverage of all major metabolite classes that could characterize the plant adaptation to the environmental changes, resulting in the annotation of 280 metabolites (Supplementary Tables 3, 4, 5, 6, 7 and 8); 215 were detected in the leaves and 195 in the roots.

**Figure 6:**
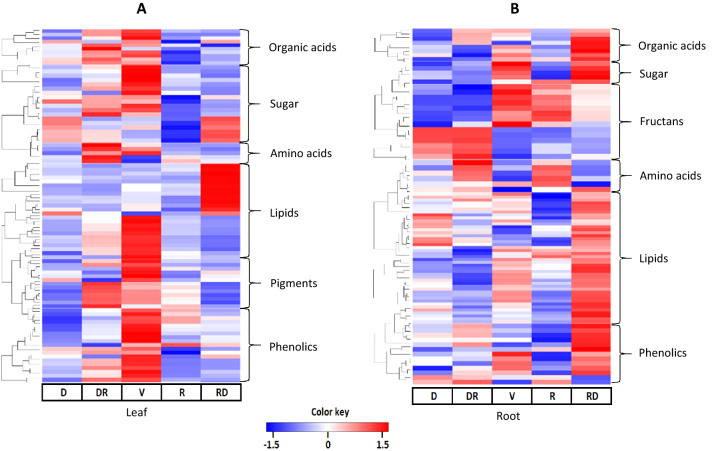
Heatmap visualization from a cluster hierarchical analysis of annotated metabolites of leaf and root of *Gomphrena agrestis* Mart. in a rock-field area in the dry and rainy seasons. *D* dry period, *RD* transition from dry to rainy period, *V* veranico period, *R* rainy period, *RD* transition from rainy to dry period. Performed using R-software version 3.4.1^66^.

For better interpretation, the annotated metabolites were clustered using a non-hierarchical analysis according to their metabolite class (Fig. 6). As expected, different metabolite patterns were observed in the leaves and roots of *G. agrestis*. The analysis of the leaves showed a unique pattern for Veranico samples, and the accumulation of organic acids, sugars, galactolipids, phenolics, and chlorophyll degradation products were observed. Interestingly, contrasting accumulation of some sugars, phosphocholine (PC), triacylglycerolipids (TAG), and galactolipids (MGDG and DGDG) were verified between Veranico and RD. Accumulation of amino acids and pigments (chlorophylls and ketocarotenoids) was observed in the leaves during the transition from drought to rainy season. In the roots, a distinct fructan pattern was observed: plants grown during Veranico and rainy accumulated fructans containing less than 10 units of fructose, and plants grown during dry and DR accumulated more complex fructans. In general, higher levels of amino acids were observed in the DR and R seasons.

### Drought-discriminating metabolites

**Figure 7:**
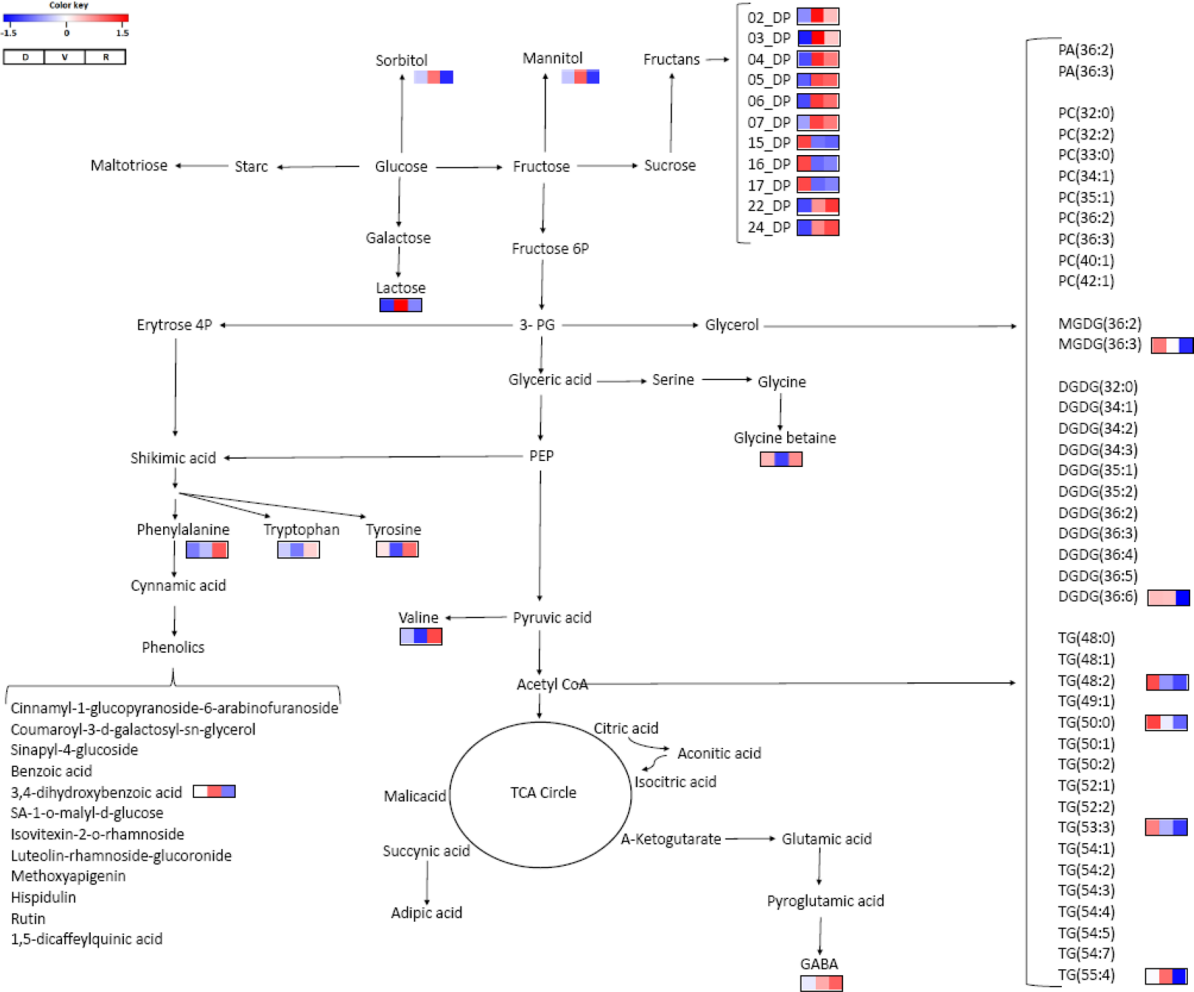
Metabolic pathway indicating the annotated metabolites of *Gomphrena agrestis* Mart. roots in a rock-field area in the dry and rainy seasons. Bars indicate relative intensity of metabolites, with pairwise comparisons of higher changes between the sample groups R × D and R × V. *D* dry period, *V* veranico period, *R* rainy period. *DP* degree of polymerization − number of fructose residues in the molecule.

**Figure 8:**
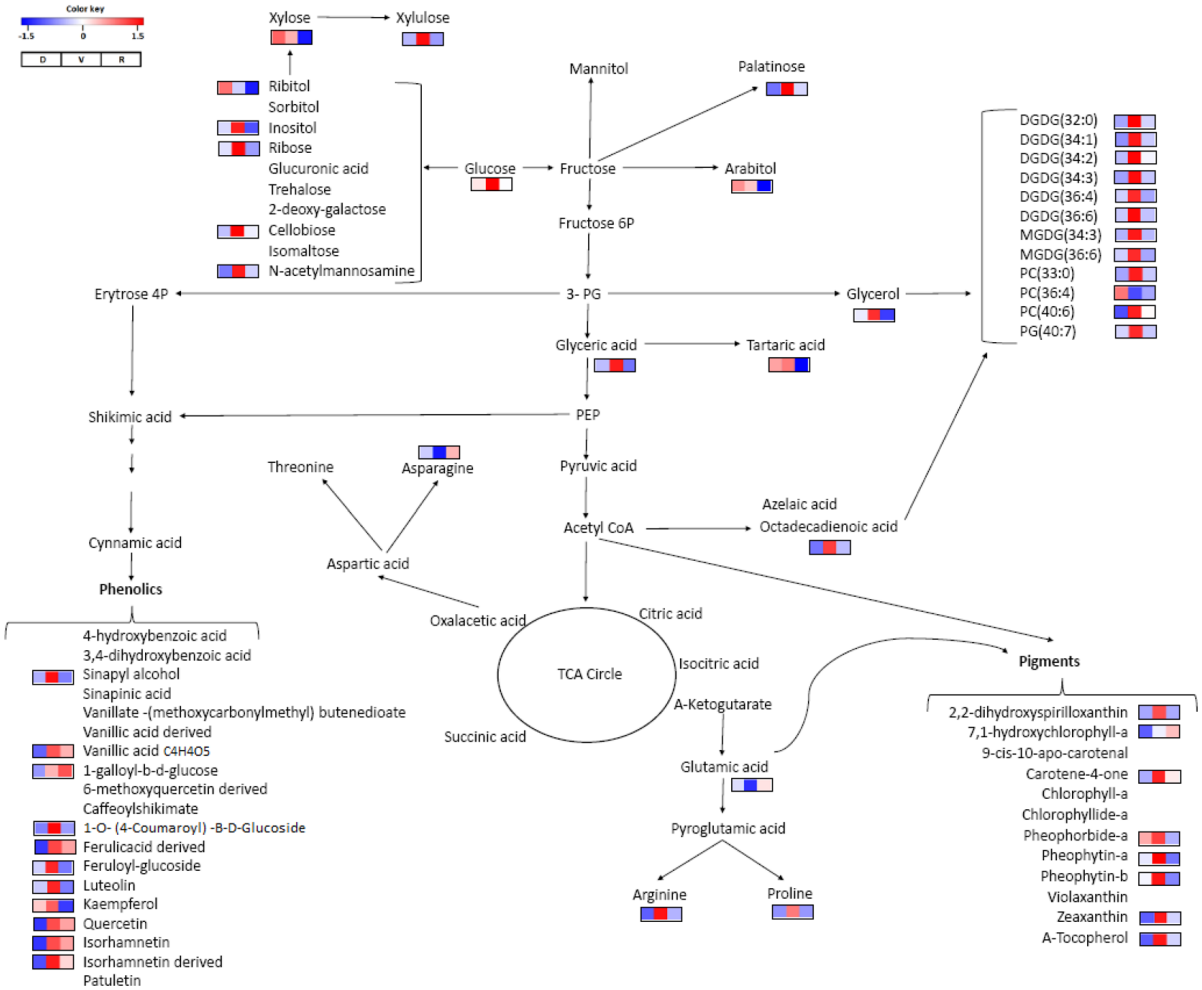
Metabolic pathway indicating the annotated metabolites of *Gomphrena agrestis* Mart. leaves in a rock-field area in the dry and rainy seasons. Bars indicate relative intensity of metabolites, with pairwise comparisons of higher changes between the sample groups R × D and R × V. *D* dry period, *V* veranico period, *R* rainy period.

The impact of water availability in the soil on the leaves and roots of *G. agrestis* grown in the rainy, dry, and Veranico seasons is shown in Figs. 7 and 8 and Supplementary Tables 9 and 10. In general, changes in the metabolism of sugar, specially fructans, lipids, amino acids, and phenolics were observed in the roots. The accumulation of fructans containing 15_DP, 16_DP, and 17_DP units of fructose and glycerolipids MGDG (36:3), DGDG (36:5), PC (33:0), TG (50:2), TG (48:2), and TG (53:3) was pronounced during the dry season, which is in contrast to the reduced levels of fructans containing small units of fructose and amino acids GABA, phenylalanine, tyrosine, and valine during the rainy (R) season. However, during Veranico, accumulation of the sugars lactose, sorbitol, and mannitol, fructans 2_DP and 3_DP, lipid TG (55:4), and the phenolic 3,4-dihydroxybenzoic acid was observed, and a decrease in the amino acids valine, phenylalanine, tryptophan, and glycine betaine was observed. The metabolism of sugars, lipids, phenolics, and pigments was affected in the leaves in the dry, Veranico, and rainy seasons (Fig. 8), with pronounced accumulation of galactolipids, xanthophylls, chlorophyll intermediates, and several classes of phenolics such as phenylpropanoids, benzoic acid, and flavonoids in Veranico. Although the dry and Veranico seasons were characterized by low water availability in the soil, the metabolism of *G. agrestis* responded differently.

## Discussion

In the Brazilian Cerrado, the vegetation is adapted to a seasonal climate with short and long periods of drought. This condition causes the local species to experience low water availability during specific periods of the year. In the present study, the water status in the soil of a rock-field area in the Cerrado changed from low to high moisture, which directly impacted the water status of plants collected from the same area during different months of the year (Figs. 1 and 3). Endemic species, like *Gomphrena*, in the Brazilian Cerrado are adapted to such water variations and can survive via different and perhaps unknown physiological strategies. In general, plant adaptation can include changes in the developmental stage and phenology of plants obtained during different seasons (Fig. 2).

The global analytical approach used to analyze *G. agrestis* allowed us to obtain a unique metabolite fingerprint of plants growing under natural conditions in Cerrado’s dry (D: July and September), dry-rainy (DR: end of September and October), Veranico (V: February), rainy (R: March and April), and rainy-dry (RD: May and June) seasons. The results provide several hints about how this endemic specie can tolerate such drastic changes in the soil water availability throughout the year. The drought stress on the plants occur when either the water supply to their roots becomes limited or when the transpiration rate becomes too intense and causes a system imbalance^1^. In both situations, the stress starts to affect the water functions, culminating in reduced growth. Here, the leaves and roots of *G. agrestis* showed reduced RWC during Veranico, resulting in an increase in ABA content (Fig. 3B). The results suggest that *G. agrestis* controls the stomatal movement as a strategy to keep the tissues hydrated or maintain the CO_2_ supply for photosynthesis, as observed in many other species^32–36^. The maintenance of open stomata can lead to a greater loss of water and consequently dehydration. However, if the soil is sufficiently moist or if the moisture is recovered soon, it is easy for the plant to replenish water and maintain both photosynthesis and the growth rate. Because of the physical characteristics of the rock-field soil (shallow and sandy) and lower precipitation rates during the Veranico period, the soil moisture decreases quickly and probably limits the water availability for the plants (Fig. 3C). ABA has an important role under stress conditions, especially during drought^6,37,38^. In the present study, we found a pronounced increase in ABA levels in the leaves during Veranico.

Generally, the root system is affected to the greatest extent when there is water scarcity or the availability of water is inconsistent^39^. The fact that the ABA levels were not significant in the roots suggest that *Gomphrena* plants have other alternatives to compensate for the drought. Fructans in the tissues may act as osmotic solutes to maintain the water status of tissues^28^. Our results support their suggestion because we found increased levels of fructans containing up to 8 units of fructose during the Veranico season (Fig. 7). Similar results were obtained by^40^, who reported no changes in the RWC in the roots of *Gomphrena marginata* (also growing in a rock field) during the dry period, suggesting that accumulation of fructans could result in osmoregulation. Similar results were found in *Vernonia herbacea*, another local species present in the rock fields of Cerrado^29,41^. The dry season (during which the soil moisture levels were lower; Fig. 3C) was characterized by the accumulation of more complex fructans (Fig. 7). In general, complex fructans (containing up to 22 fructose units) were higher during the dry and DR periods. Fructans with lower DP were predominant during the Veranico and rainy periods (Figs. 6B and 7). These contrasting patterns are consistent with the involvement of fructans in drought strategies: complex fructans represent a carbon source that supports initial growth or regrowth during the beginning of the rainy season^26,40^. Simple fructans in the rainy and Veranico periods can be explained by the water-favorable condition for synthesis and turnover of the energy metabolism in the rainy season or a strategy to support osmoregulation during the Veranico.

The accumulation of sugar alcohols such as arabitol and ribitol in the leaves during the dry season (Fig. 8) suggests their involvement in the non-photochemical quenching during drought^42^. However, other simple sugars (e.g., glucose, fructose) may also play a role in drought tolerance in plants by reducing the effects of osmotic stress, maintaining turgor, stabilizing cell membranes, and protecting plants from damage^43^. The level of xylose was also high in the dry season, and it is a component of cell wall metabolism and suggested to be involved in drought stress through cell wall modification^35^.

Accumulation of phenolic metabolites during the dry and Veranico seasons was observed. The phenylpropanoid pathway was more pronounced during Veranico, resulting in the accumulation of sinapinic acid, sinapyl alcohol, 1-O-(4-coumaroyl)-B-d-glucoside, feruloyl-glucoside, and caffeoylshikimate as well as different flavonoids, which is in contrast to the accumulation of benzoic acid derivatives like vanillic acid and 1-galloyl b-d-glucose during the dry season (Fig. 8). Such accumulation of phenolic metabolites in both seasons might be related to plant protection against oxidative damage that occurs during these two periods of low water availability^21,45^. The production of reactive oxygen species may be the most important secondary effect of drought and can result in chloroplast membrane damage. We observed the accumulation of several galactolipids during Veranico. These lipids are major components of the photosynthetic apparatus^46^.

Pigments like carotenes can act as antioxidants and energy quenchers^47^. Carotenes and xanthophylls, as well as chlorophyll metabolites like chlorophyllide, pheophorbide and pheophytin, were accumulated in the plants during Veranico (Fig. 8). Pheophytin is involved in the process of electron transfer in PS II, working as a bridge of electrons between the chlorophyll P680 and plastoquinone^48,49^. Previous studies have investigated how this mechanism works and the function of pheophytin^50,51^. In this study, the increased levels of pheophytin may be due to induced chlorophyll degradation^52^, the stress, or a response that benefits the plant. However, no chlorophyll changes were observed in the plants collected during Veranico. Therefore, pheophytin accumulation may be a mechanism that helps in photosynthesis efficiency by either acting in the flux of electrons or protecting the system from damage^50,53^. The role of pheophytin in plants is not well understood and needs to be studied under stress conditions.

It is important for a plant to adapt to yearly (long-term) and short-term changes in drought and other environmental factor to coordinate growth and stress-related responses. Stomatal closure acts by reducing the loss of water and maintaining the hydration state in the tissues, but it also limits CO_2_ influx for photosynthesis. *G. agrestis* is a C4 plant^54^ and therefore exhibits efficient photosynthetic metabolism under drought conditions. Probably the plant showed high photosynthesis rates in the Veranico period, even with stomatal control. This strategy is important under mild or short-term water stress conditions because the plant can sustain growth, which is primarily affected. The adaptions of metabolism, as observed in the present study, suggests strategies to maintain photosynthesis during the Veranico period. This is of great importance to *G. agrestis* because this period coincides with the flowering time (Fig. 2), which requires high quantities of assimilated carbon. The minimum hydration necessary for survival, cell enlargement, or maintenance of metabolic activity is provided by regulated stomata control and other associated strategies, such as osmoregulation.

## Conclusions

In this study, we used a metabolomic approach to understand and describe the metabolic adaption of a native species to seasonal changes in drought. We showed that fructans are accumulated in the thickened roots, suggesting a metabolic pattern that is consistent with the storage of carbons during the water-favorable season to support energy demand during the long period of drought and regrowth as well as metabolic adjustments for osmoregulation. In the leaves, ABA, simple sugars, sugar alcohols, phenolics, and pigment metabolism indicate the importance of metabolic responses that should act together to modulate general physiological adaptations such as stomatal control, photosynthesis, and oxidative stress. The metabolic pattern in the Veranico period suggests that during short-term drought, the maintenance of active photosynthesis seems to be more important, and stomatal control, osmoregulation, and protection from oxidative damage may be the strategies used by the species.

## Methods

### Geographical location

The study was conducted in the Environmental Preservation Area “Serra do Resplandecente Encantado”, a public area in the municipality of Itacambira, north of Minas Gerais State (16° 59′ 47″ S, 43° 20′ 01″ W), Brazil. In this area, which is part of the Espinhaço mountain range, rock-field formations are predominant.

### Plant material and sampling

The study was conducted in accordance with relevant guidelindes and brazilian legislation^55^. Plant collection was registered in SisGen (Sistema Nacional de Gestão do Patrimônio Genético e do Conhecimento Tradicional) with registration code AFBE204, in October 2018. Specimens of *G. agrestis* Mart. were collected, properly identified by the PhD Maria Salete Marchioretto, herborized and deposited (voucher number—PACA 114593) at Herbarium Anchieta/PACA (Anchietano Research Instiutute/UNISINOS, São Leopoldo- RS, Brazil). The sampling was conducted in two periods to understand and describe the transition between the dry and rainy seasons. The first period was from September to November 2013, which includes the end of the dry season and beginning of the rainy season, and the second period was from February in the rainy season to July 2014, which already corresponds to the dry season. Sixteen field expeditions were conducted, and six plants (of similar size and development stage) were collected each time, totalizing 96 individual plants sampled. The plants were conditioned in plastic bags and maintained in a Styrofoam box prior to transport to the laboratory. The leaves and roots were immediately frozen in liquid nitrogen, ground, freeze-dried, and stored in a freezer (− 20 °C) until analysis. The climate data were obtained from the National Institute of Meteorology (INMET), Brazil (http://www.inmet.gov.br), to characterize the local climate during the sampling. The study was conduct on a single year because later studies^22,41^ in the area/region has shown stability and reproducibility of the data along the years.

### Phenology annotation

In each expedition, 30 plant individuals were randomly photographed, and each photograph was analyzed to identify the presence or absence of phenological stages: (1) sprouting, emission of new branches and leaves; (2) flowering, presence of floral branches and inflorescences with reddish floral parts; (3) fruiting, presence of inflorescences with senescent floral parts and paleaceous coloration; and (4) dormancy, presence of floral branches and older leaves of the branch in senescence.

### Relative water content

To characterize the water status of the plants, 10 leaves and root fragments were collected from each plant and weighed for determining the fresh weight (FW). Then, they were immersed in distilled water for 6 h to determine the turgid weight (TW), followed by drying at 70 °C to determine the dry weight (DW). The relative water content (RWC) was estimated using the following equation: RWC (%) = [(FW − DW)/(TW − DW)] × 100^56^.

### Soil moisture

Soil moisture (Sm, %) was measured using the gravimetric method^57^. In each field expedition, six soil samples were collected between 0 and 20 cm, which corresponds to the effective root depth. The soil samples were weighed to determine FW and then dried at 70 °C to measure DW. Sm (%) was determined using the following formula: Sm (%) = (FW − DW/DW) × 100.

### Metabolite measurements

Leaves and roots from 11 expeditions (66 plants) were selected (Fig. 1) for the metabolite profiling by using different approaches such as untargeted metabolomic (GC-QTOF MS and LC-QTOF MS) and lipidomic analyses (LC-Qtof MS) and targeted (LC-QqQ MS) analysis of amino acids, fructans, and abscisic acid (ABA).

#### Extractions

For the untargeted metabolomic and amino acid analysis, 10 mg of dried and ground leaves and roots were extracted^58^. For the lipidomic analysis, 10 mg of the dried samples was extracted with 500 µL of chloroform:methanol (2:1, v/v). After vigorous shaking, 100 µL of NaCl (0.15 M) was added and let to stand for 30 min for phase separation and then centrifuged (14,000 rpm for 3 min). The chloroform phase was collected for analysis. For fructans, 20 mg of the samples was extracted with 500 µL of boiling Milli-Q water. After 15 min at 90 °C, the extracts were cooled down to room temperature and centrifuged (14,000 rpm for 2 min). The supernatant was collected and filtered with 22 micron filters and immediately used for analysis. ABA was extracted from 20 mg of the samples in 1 mL of 80% methanol, containing 1 pg/µL of deuterated abscisic acid (d6-ABA), purchased from OlChemIm (Olomouc, Czech Republic). After vigorous shaking, the samples were centrifuged (14,000 rpm for 10 min), and the supernatant was collected and vacuum-dried. Then, the pellets were recovered in 200 µL of 10% methanol (containing 1% acetic acid) and purified in SPE columns (Oasis HLB, 30 µm—Waters).

#### Untargeted metabolomic analysis with GC-TOF MS

The extracts were derivatized overnight in room temperature with 30 μL methoxyamine (15 ng/μL pyridine), and thereafter with 30 μL MSTFA with 1% TMCS for 1 h in room temperature for 1 h. 30 μL of methyl stearate (15 ng/μL in heptane) were added before analysis. 0.5 μL of the derivatized sample was injected in splitless mode by a L-PAL3 autosampler (CTC Analytics AG, Switzerland) into an Agilent 7890B gas chromatograph equipped with a 10 m × 0.18 mm fused silica capillary column with a chemically bonded 0.18 μm Rxi-5 Sil MS stationary phase (Restek Corporation, U.S.) The injector temperature was 270 °C, the purge flow rate was 20 mL/min and the purge was turned on after 60 s. The gas flow rate through the column was 1 mL/min, the column temperature was held at 70 °C for 2 min, then increased by 40 °C/min to 320 °C, and held there for 2 min. The column effluent was introduced into the ion source of a Pegasus BT time-of-flight mass spectrometer, GC/TOFMS (Leco Corp., St Joseph, MI, USA). The transfer line and the ion source temperatures were 250 °C and 200 °C, respectively. Ions were generated by a 70 eV electron beam at an ionization current of 2.0 mA, and 30 spectra/s were recorded in the mass range *m/z* 50–800. The acceleration voltage was turned on after a solvent delay of 150 s. In addition an alkane mixture (C12–C40) was analysed to determine the retention indices of the detected compounds^59^. The generated spectral files were converted to NetCDF format and processed using *in-house* scripts for MATLAB ver. 8.1 (Mathworks, Natwick, MA, USA). The detected peaks were identified by comparison of mass spectra and retention indexes using NIST MS Search v.2.0 search by in-house and NIST98 spectral databases.

#### Untargeted metabolomic and fructans analyses with LC-QTOF MS

The chromatographic separation for untargeted and fructans LC–MS analyses^60^ was performed on an Agilent 1290 Infinity UHPLC-system (Agilent Technologies, Waldbronn, Germany). 2 µL of re-suspended aliquots of extracted plasma or plant sample were injected onto an Acquity UPLC HSS T3, 2.1 × 50 mm, 1.8 µm C18 column in combination with a 2.1 mm × 5 mm, 1.8 µm VanGuard precolumn (Waters Corporation, Milford, MA, USA) held at 40 °C. The gradient elution buffers were A (H2O, 0.1% formic acid) and B (75/25 acetonitrile: 2-propanol, 0.1% formic acid), and the flow-rate was 0.5 mL/min. The compounds were eluted with a linear gradient consisting of 0.1–10% B over 2 min, B was increased to 99% over 5 min and held at 99% for 2 min; B was decreased to 0.1% for 0.3 min and the flow-rate was increased to 0.8 mL/min for 0.5 min; these conditions were held for 0.9 min, after which the flow-rate was reduced to 0.5 mL/min for 0.1 min before the next injection.

The compounds were detected with an Agilent 6550 Q-TOF mass spectrometer equipped with a jet stream electrospray ion source operating in positive or negative ion mode. The settings were kept identical between the modes, with exception of the capillary voltage. A reference interface was connected for accurate mass measurements; the reference ions purine (4 µM) and HP-0921 (Hexakis (1H, 1H, 3H-tetrafluoropropoxy phosphazine) (1 µM) were infused directly into the MS at a flow rate of 0.05 mL/min for internal calibration, and the monitored ions were purine *m/z* 121.05 and *m/z* 119.03632; HP-0921 *m/z* 922.0098 and *m/z* 966.000725 for positive and negative mode respectively. The gas temperature was set to 150 °C, the drying gas flow to 16 L/min and the nebulizer pressure 35 psig. The sheath gas temp was set to 350 °C and the sheath gas flow 11 L/min. The capillary voltage was set to 4000 V in positive ion mode, and to 4000 V in negative ion mode. The nozzle voltage was 300 V. The fragmentor voltage was 380 V, the skimmer 45 V and the OCT 1 RF Vpp 750 V. The collision energy was set to 0 V. The *m/z* range was 70–1700, and data was collected in centroid mode with an acquisition rate of 4 scans/s (1977 transients/spectrum). For metabolite annotation autoMSMS was performed on pooled QC-samples at 3 different collision energies, 10, 20 and 40 eV.

The fructans compounds were detected with an Agilent 6550 QTOF mass spectrometer equipped with a Jet Stream electrospray ion source operating in positive and negative ion mode^60^. The MS/MS spectra were obtained under the same conditions, with the collision energy from 10 to 40 V.

All generated files were processed using Profinder B.08.00 (Agilent Technologies). The metabolomic data have been deposited to the EMBL-EBI MetaboLights public repository (https://www.ebi.ac.uk/metabolights/MTBLS613).

#### Lipidomic analysis with LC-QTOF MS

The lipid analysis was performed in the positive ion mode^61^. In brief, lipid extracts based on chloroform/methanol extraction was chromatographic separation was performed on an Acquity UPLC CSH, 2.1 × 50 mm, 1.7 µm C18 column in combination with a 2.1 mm × 5 mm, 1.7 µm VanGuard precolumn (Waters Corporation, Milford, MA, USA) held at 60 °C. The gradient elution buffers were A (60:40 acetonitrile:water, 10 mM ammonium formate, 0.1% formic acid) and B (89.1:10.5:0.4 2-propanol:acetonitrile:water, 10 mM ammonium formate, 0.1% formic acid), and the flow-rate was 0.5 mL/min. The compounds were detected with an Agilent 6550 Q-TOF mass spectrometer equipped with a jet stream electrospray ion source operating in positive ion mode. All mass spectrometer settings as for untargeted LC–MS analysis. All generated files were processed using Profinder B.08.00 (Agilent Technologies).

#### Metabolite annotation

The metabolites were annotated by manual interpretation of the high mass accuracy of the fragments produced by MS/MS experiments and/or comparison with public (Kegg and PlantCyc) and in house database. Additional MS/MS networking (Global Natural products social molecular networking^62^) was performed as a quality control to detect adduct masses that somehow were not excluded during the processing data. For annotation of fructans, the degree of polymerization, which means the number of fructose units in the molecule structure was used. Glycerolipids annotation was performed by comparison with in house lipid spectral databases. The lipid classes were differentiated by the presence of diagnostic fragments *m/z* 184.0733 (PC), *m/z* 243.0945 (MGDG-Na^+^) or neutral losses of 162.0528 (DGDG), 161.0450 (PI) and 141.0191 (PE). Spectral information of phenolics and lipids are presented in Supplementary Tables 5 and 7.

#### Amino acid analysis with LC-QqQ MS

The extracts were derivatized with the Waters AccQ•Tag method, in accordance with the manufacturer’s protocol. The analysis was performed using a 1290 Infinitely UHPLC system from Agilent Technologies (Waldbronn, Germany) with G4220A binary pump, G1316C thermostated column compartment, and G4226A autosampler with G1330B autosampler thermostat coupled to an Agilent 6490 triple quadrupole mass spectrometer equipped with a jet stream electrospray source operating in the positive ion mode^63^. The amino acid multiple-reaction-monitoring (MRM) transitions were optimized using MassHunter MS Optimizer software (Agilent Technologies Inc., Santa Clara, CA, USA), and the data were quantified using MassHunter Quantitation software B07.01 (Agilent Technologies); the amount of each amino acid was calculated on the basis of the calibration curves.

#### ABA analysis with LC-QqQ MS

For the ABA analysis^64^ the analytes were separated using a 1290 UHPLC system from Agilent Technologies (Waldbronn, Germany), with a G4220A binary pump, G1316C thermostated column compartment, and G4226A autosampler with thermostat. A 2 µL aliquot of the sample was injected onto a Waters column (TSS3, C18; 2.1 × 50 mm, 1.7 µm) at 40 °C in a column oven. The analysis was performed in multiple-reaction-monitoring (MRM) mode, in which the fragmentation conditions for the analyses were optimized using MassHunter MS Optimizer software (Agilent Technologies Inc., Santa Clara, CA, USA). MRM scan was performed monitoring *m/z* 263 → 153 for ABA and m/z 269 → 159 for d6-ABA as quantifiers. Transitions *m/z* 263 → 219 for ABA and *m/z* 269 → 225 for d6-ABA were used as qualifier ions. The data were quantified using MassHunter Quantitation software B07.01 (Agilent Technologies); the amount of ABA was calculated on the basis of the calibration curve done with d6-ABA (1 pg/µL) and ABA standards (from 0 to 10 pg).

### Statistical analysis

The generated datasets from the different analyses were checked using statistical multivariate analysis in SIMCA-P 13 software (Umetrics AB, Umeå, Sweden). The samples were compared using PCA and OPLS-DA analysis. Before the analysis, the missing data were set to the mean value of each variable and were mean-centered and scaled to the unit variance. The samples were grouped according to the environment characterization into five groups: 1, dry (D: 12 samples); 2, transition between dry to rainy (DR: 18 samples); 3, “Veranico”, a short dry period during the rainy season (V: 6 samples); 4, rainy (R: 12 samples); and 5, transition between rainy to dry (RD: 18 samples). To identify the most important metabolites in the OPLS-DA models, the VIP was used, and variables showing VIP values greater than 1 were considered of high importance^65^. The OPLS-DA models were validated using the goodness of fit (R^2^) and prediction (Q2) parameters. Further statistical analysis and visualization (ANOVA, Tukey’s test, *t*-test, Benjamini and Hochberg correction, and heatmaps) were performed using R-software version 3.4.1^66^.

## Supplementary Information

Supplementary Table S1.

Supplementary Table S2.

Supplementary Table S3.

Supplementary Table S4.

Supplementary Table S5.

Supplementary Table S6.

Supplementary Table S7.

Supplementary Table S8.

Supplementary Table S9.

Supplementary Table S10.
